# Progress and Challenges in Joining for Precision Endoscope Fabrication

**DOI:** 10.3390/s26092828

**Published:** 2026-05-01

**Authors:** Peiquan Xu, Xiaohao Zheng, Leijun Li, Ziyi Wang

**Affiliations:** 1 School of Materials Science and Engineering, Shanghai University of Engineering Science, Shanghai 201620, China; zxh1347342205@outlook.com; 2 Department of Chemical and Materials Engineering, University of Alberta, Edmonton, AB T6G 1H9, Canada; 3 School of Integrated Circuits, Chongqing University of Posts and Telecommunications, Chongqing 400065, China; 2023215081@stu.cqupt.edu.cn

**Keywords:** endoscope, minimally invasive surgery (MIS), welding, joining, sealing

## Abstract

This review summarizes the base materials, joining methods, filler materials, and principal technical challenges in endoscope joining fabrication, and proposes practical strategies to improve joint reliability under clinical constraints. We conducted a comprehensive search in multiple databases, including Web of Science, Google Scholar, patent databases, Scopus databases, and Medline (via PubMed), for articles on the joining for precision endoscope fabrication, covering the period from 1950 to 2026. We employed the combinations of keywords, “endoscopy”, “minimally invasive surgery”, “welding”, “joining”, “sealing”, “soldering”, “bonding”, and “brazing”. Approximately 500 references were retrieved. After excluding duplicates and irrelevant studies, 158 publications met the inclusion criteria. Data on base materials, joining, processes, filler materials, and technical issues related to sterilization, corrosion, and microstructural evolution were extracted and analyzed. Endoscopes are multi-material systems, involving metallic biomaterials (stainless steels (SSs), titanium alloys, nickel-based alloys, etc.), optical functional materials (glass, sapphire, quartz, etc.), engineering plastics, ceramics, composite materials, and coatings. Joining, sealing, and functional integration have been achieved via adhesive bonding, laser soldering, laser brazing, wave soldering, reflow soldering, fusion welding, and other joining techniques. The main challenges include how to reliably join highly mismatched dissimilar materials, how to fabricate low-residual-stress joints, and how to increase the long-term resistance to sterilization-induced degradation and thermal aging over repeated 100–200 °C thermal cycles. Conventional joining techniques struggle to balance mechanical integrity, joint hermeticity, and long-term stability under such harsh cyclic conditions. The resulting joints may suffer surface yellowing, interfacial debonding, microcracking, delamination, or progressive property degradation during service. We propose the following three strategies to achieve reliable, low-residual-stress, and sterilization-resistant joining of dissimilar materials for endoscopes: (1) A synergistic design that combines thin-film engineering (including evaporation, sputtering, and electroplating) with silver anti-oxidation layers is proposed to reduce residual stresses and to enhance the joint hermeticity. (2) To develop principles for the selection of multi-joining processes to achieve the multi-material integration and functional assembly of dissimilar material components. (3) To develop the laser-based joining methods (fusion, brazing, or braze-welding) for precision control of heat input, bonding quality, and the least damage to the heat-sensitive components.

## 1. Introduction

One of the most important characteristics and advances in modern medicine is the direct exploration of organs to elucidate their physiology and pathology [1]. The invention of the otoscope, ophthalmoscope, laryngoscope, and endoscope has greatly improved doctors’ ability to treat intractable and complex diseases.

Compared with traditional diagnostic and detection technologies, endoscopy offers the following advantages: minimally invasive nature [2,3,4,5,6], real-time imaging [7,8], rapid diagnosis [9,10], broad applicability [11,12,13], comprehensive lesion evaluation [14], and reduced hospitalization duration.

Endoscopy uses small incisions or natural orifices, greatly reducing trauma and recovery time. It delivers real-time, high-resolution images for rapid intraoperative lesion assessment, allows immediate biopsy or treatment, and is applicable to many sites (digestive, respiratory, urinary). By enabling intuitive observation of lesion morphology and earlier discharge, it improves diagnostic accuracy and lowers hospitalization costs.

An endoscope, as an imaging device, consists of a light source [15,16,17], optical lens [18,19], optical cable, an image transmission system [20,21], a screen display system [22,23], and mechanical devices [24,25,26]. The mechanical system of an endoscope comprises an insertion tube assembly, a bending mechanism, a control handle, a distal end assembly, and an auxiliary mechanical system [27,28]. It is structured around the framework of the insertion tube, bending mechanism, and control handle, with the instrument channel serving as a functional extension, supplemented by auxiliary modules such as air/water delivery and sealing. Its design essence lies in balancing mechanical performance (flexibility, rigidity, durability) and biocompatibility (low trauma, miniaturization, and disinfection resistance) [29,30]. Endoscopes have attracted wide research interest in optimizing their performance and reliability [25,31,32,33].

The rapid advancement of endoscope fabrication has effectively surmounted the long-standing challenge that physicians could not directly visualize pathological organs inside the human body—an obstacle that has long impeded precise diagnosis and targeted therapy. Nevertheless, notwithstanding these remarkable technological breakthroughs, inherent deficiencies persist in the design and fabrication of endoscopes, which may introduce potential safety hazards for both patients and clinicians.

Notably, joining methods (e.g., brazing [34] or welding) are critical to endoscope performance: seal quality determines resistance to bodily fluids and sterilization, joint strength affects the device’s structural lifetime, and biocompatibility and miniaturization suitability ensure patient safety and minimally invasive operation. The related research includes laser welding of folded plastic layers [35], sapphire/steel bonding [36], quartz–SS joints [37], sapphire/Fe–36Ni joints [38], G3.3/SnAgCu-xSc/316L SS [39], pressure sensor consisting of a Pyrex glass tube sealed to a titanium adapter joining to a ventricular catheter [40], and regenerated oxidized cellulose in endoscopic endonasal reconstruction [41], among others. However, most existing studies only focus on joining techniques in isolation, lacking systematic comparisons for endoscope fabrication, and fail to establish a clear correlation framework among material, joining processes, and clinical requirements.

The aim of the review is to clarify the joining processes, their applicable material systems, and the corresponding market-oriented selection strategies for clinical applications. It focuses exclusively on joining/joining technologies in endoscopic device manufacturing rather than providing a general overview of endoscopic system development

This review systematically summarizes the technical characteristics, applicable scenarios, and material compatibility of typical joining in endoscope fabrication. It clarifies the matching relationship between joining processes, base materials, and clinical performance, and proposes rational selection routes and future work for high-reliability and miniaturized endoscopic joining technologies.

## 2. Endoscopes, Sensors, and Their Materials for Precision Joining

**Figure 1:**
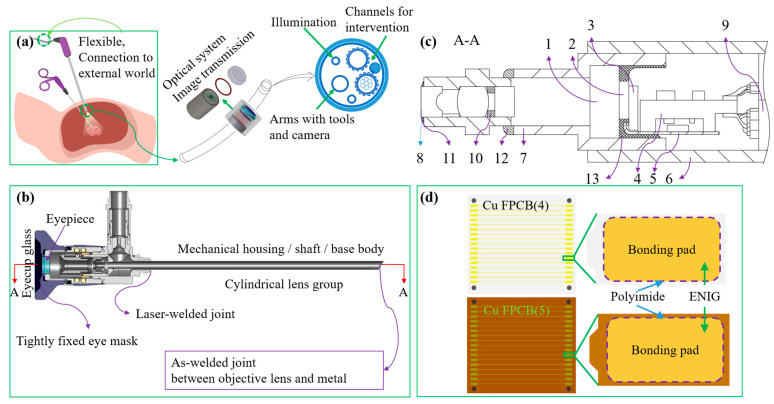
Schematic diagrams of the endoscopic system and its structural details. (**a**) An endoscope in clinical application: depiction of a laparoscopic MIS into the body cavity [42]; inset shows a distal tip with multiple channels for optical system, image transmission module, illumination channel, and intervention channels. (**b**) Cross-sectional view of a rigid endoscope, highlighting the tightly fit eyecup/eye mask, laser-welded joints, mechanical housing/shaft/base body, cylindrical lens, and the as-welded joint between the objective lens and metal components [43]. (**c**) Enlarged axial cross-section (section A–A) of the distal module showing internal components, optical pathways, and image transmission: 1, 2—Glass covers; 3—Image sensor; 4, 5—Circuit boards; 6—Outer tube; 7—Inner tube; 8—Cover; 9—Composite cable; 10—Glass/glass adhesives; 11—Glass/metal seals; 12—Metal/metal welds; 13—Glass/PCB seal. (**d**) Schematic of the Cu/FPCB (flexible printed circuit board) bonding structure between circuit boards in (**c**), illustrating the bonding pads, polyimide layer, and ENIG (electroless nickel immersion gold) coating for electrical interconnection.

As shown in Figure 1, the structural design of the endoscope is centered on the needs of minimally invasive clinical diagnosis and treatment. Figure 1a illustrates the clinical application scenario of the endoscope in laparoscopic surgery [42]. Figure 1b presents the overall structure of the rigid endoscope, highlighting the sealing and joining of laser welding between the lens–metal component and the endoscope body assembly [43]. Figure 1c shows a cross-section of the distal imaging module, including such components as image sensor, flexible circuitry, and sealing. Figure 1d clarifies the electroless nickel immersion gold (ENIG) bonding of the flexible printed circuit board (PCB), providing key support for the high-reliability electrical joining.

From a joining and welding perspective, the scope base material refers to the totality of materials used to fabricate the various components of an endoscope. These include supporting tubes for endoscopes, protective tubes and housings, glass used as transparent windows to transmit light and isolate corrosive body fluids from endoscope components, optical fibers for cold light delivery, imaging systems for capturing and transmitting disease-related images, as well as precision instruments such as forceps, microscissors, and needles.

As shown in Table 1, base materials can be divided into three categories [44,45,46,47,48,49,50,51,52,53,54,55,56,57,58,59,60,61,62,63,64,65,66,67,68,69,70,71]: metallic biomaterials, optical functional materials (OFMs), and engineering plastic materials. No single material can satisfy all the requirements of endoscopes. Successful device design requires the integration of materials with complementary properties, rather than optimizing individual components in isolation. Material performance must be paired with scalable manufacturing processes, such as adhesive curing, microstructure control, and welding, to enable commercialization. Bodily fluids, disinfectants, and repeated sterilization create extreme operating conditions, making corrosion resistance and biocompatibility primary selection criteria, rather than secondary considerations.

In addition to the materials listed below, endoscopes also incorporate ceramic, rubber, and elastomer composites, along with polymer coatings.

### 2.1. Metallic Biomaterials

Metallic biomaterials, mainly used as structural parts, include stainless steel [44,45,46,47,48,49,50], titanium alloys [51,52,53,54,55], Monel [56], shape memory alloys (SMA) [50], Co-Cr (CC) [57,58], Ni-Co-Cr [59], and aluminum alloys [60].

Stainless steels (SSs) are among the most widely used biomaterials in endoscope systems due to their good corrosion resistance, mechanical strength, machinability, and cost-effectiveness. They are suitable for non-optical components, which require repeated disinfection and carry mechanical loads, such as the outer sleeve and sheath of the insertion part [44,45], operating and transmission structures [46], surgical accessories [47], and support and protective structures for optical components [48], and external connections and auxiliary components. Type 316L, although commonly used in endoscopes, does not possess inherent antibacterial properties, and the release of nickel ions may trigger hypersensitivity reactions in some individuals. In contrast, Type 17-4PH [49] exhibits intrinsic antibacterial capability and excellent mechanical properties, making it a promising candidate for medical applications.

Titanium alloys, including nickel–titanium shape memory alloys, are mainly used in the core structure of the insertion section, operational and imaging components, and surgical accessories. Their advantages include low density, high fatigue strength, high tensile strength, corrosion resistance, and good biocompatibility [50]. Titanium alloys can serve as bending components, NiTi needles, titanium blades [51], cystoscope extraction devices [52], microforceps and microscissors [53], push rods [54], flexible robotic bronchoscopic end effectors [55], traction wires, stents, housings, and other components. Their superelasticity and shape memory effect support precise steering and complex operations. Titanium alloy housings are commonly used in high-end medical camera handles and industrial endoscope probes, balancing strength and weight while being suitable for disinfection and frequent use. Disposable titanium clips and other accessories are fabricated from implant-grade titanium alloys, meeting biocompatibility requirements for both short-term and long-term implantations.

Tubular components made of brass, stainless steel, Cu-Ni-Zn alloy, or Monel [56] are essential parts in various medical devices. The use of Co-Cr (CC) for manufacturing aspiration needles also offers advantages over stainless steels [57,58]. For endoscopic hermetic sealing, our recent work developed a scandium-modified SnAgCu-*x*Sc solder for G3.3/316L brazing; Sc addition refined the microstructure, improved wettability, and numerical optimization further reduced joint residual stress [39].

### 2.2. Optical Functional Materials (OFMs)

Optical functional materials are used in cold light sources and visual imaging light path systems; they mainly include optical lens materials, such as borosilicate glass, aluminosilicate glass, quartz glass, polymethyl methacrylate (PMMA), polycarbonate (PC), cyclic olefin copolymer (COC), light guide materials, such as fused silica optical fibers, multimode optical fibers, polymer-based planar waveguides, and optical coating materials, such as silicon dioxide (SiO_2_), titanium dioxide (TiO_2_), magnesium fluoride (MgF_2_) composite films, fluoropolymer films, diamond-like carbon films (DLC), silver ion-doped silicon dioxide films, and zinc oxide nanocomposite films. Specific examples include sapphire glass [61,62], corundum [63], optical glass [64], polymethyl methacrylate (PMMA) [65], quartz fiber [66], etc.

### 2.3. Engineering Plastic Materials

Engineering plastic materials, mainly used for various flexible or bendable components and sealing components, include acrylonitrile butadiene styrene (ABS) [66], polycarbonate (PC) [67], polyether ether ketone (PEEK) [68,69], polyphenylsulfone (PPSU) [70,71] etc. Some engineering plastics can also be used for the structural components of endoscope operating handles. Such plastics include polyamide (nylon, PA), polyoxymethylene (POM), polyphenylene sulfide (PPS), polyacrylonitrile (PAN), polyethylene terephthalate (PET), polyethylene (PE), and polypropylene (PP).

## 3. Precision Joining of Endoscope Base Materials

The endoscope is a complex electromechanical device composed of numerous precision components of various materials, making the high-precision welding and joining essential for its fabrication.

### 3.1. Purpose of Joining

The purposes of joining are primarily as follows:

(1) Structural Joining. Soldering [72], brazing [73], welding (fusion welding, pressure welding [74], laser welding [75]), bonding [76], and mechanical joining (e.g., fastening, riveting, bolting, screwing, and clamping) [77] enable the formation of bonds between individual components, ensuring the structural strength and integrity of the assembled endoscope. Joining methods, such as UV-curing medical adhesives, are used for bonding the objective lens group to the CMOS/CCD sensors [78,79,80].

(2) Hermetic Sealing [72]. Seals are either welded using specialized techniques or secured with epoxy (adhesive bonding) to prevent body fluids, disinfectants, moisture, and contaminants from entering the interior, thereby protecting optical modules, sensors, and circuits.

During service, endoscopes are often used in corrosive fluid or high-humidity environments [81], exposed to disinfectant immersion or 140 °C autoclave sterilization with substantial thermal–mechanical stress [82], and prone to adhesive aging at the proximal eyepiece window during sterilization [83], all of which may lead to leakage. Seal failure can be caused by flash sterilization, shaft bending, or accidental dropping.

(3) Functional Integration: Through joining [84], various components can work together effectively, transmitting optical signals, mechanical forces, and control commands smoothly. This integration enables the endoscope to function for precise imaging, stable operation, and reliable performance in clinical applications.

In addition to the above-discussed joining, endoscope fabrication also employs additive manufacturing (3D printing) [85,86], laser micromachining, ultra-precision machining, precision injection molding [87], and surface modification. Additive manufacturing enables the integrated fabrication of complex microstructures and customized components.

### 3.2. Filler Materials

Filler materials are required for some of the joining processes. As shown in Table 2, common intermediate filler materials include: adhesives [88,89]; seals [90]; solders [91]; brazing alloys [92]; and welding electrodes, welding wires, and fluxes [93]. Some consider the shielding gases as filler materials [94,95], because they provide functions similar to fluxes.

#### 3.2.1. Adhesives

Adhesives, also known as bonding agents, are substances that can firmly join two or more materials through interfacial adhesion and cohesive. Their primary role is to replace or assist traditional methods such as welding and mechanical joining, achieving seamless bonding between components.

In biomedicine, adhesives exhibit versatile applications: they bond components of disposable products, provide long-life connections in various surgical procedures, and facilitate the assembly of endoscopes, sensors, and other biotechnological devices. However, their current use is largely restricted to non-sterilizable devices or single-use items sterilized by radiation.

Adhesives used in endoscope fabrication include (1) medical epoxy adhesives, (2) silicone adhesives, (3) UV-curable (UV) acrylic adhesives, and (4) biomedical pressure-sensitive adhesives.

Adhesives applied in endoscopes must also withstanding conventional sterilization methods, such as steam autoclaving, ethylene oxide sterilization (ETO), radiation sterilization (RS), hydrogen peroxide (H_2_O_2_) sterilization, formaldehyde (HCHO), and high-level disinfection (HLD). They should not release small molecules during curing, and the bonding interface is smooth and bubble-free.

To assess the stability and aging behavior of these adhesives under sterilization conditions, Table 3 summarizes the sterilization mode, aging, strength retention, and degradation mechanism of adhesives under repeated cycles [19,45,82,96]. It can be seen from this table that the resistance of adhesives to repeated autoclaving cycles varies with the adhesive formulation and the substrate [96]. The sterilization effects of microwave (MW), ultraviolet (UV), and gamma radiation on 3D printed implantable devices [97] confirmed that the sterilization performance of adhesives is also closely related to the sterilization mode.

Wallace outlined the advances in device sterilization in medicine [99]; however, studies on the durability of medical adhesives under repeated sterilization cycles are few, and these are listed in Table 3. The tissue adhesives are used for hemostasis, wound closure, and fistula repair [89]. It is suggested to investigate the potential adhesives, including collagen-based sealants, PEG polymers, and other formulations, in multiple surgical fields, for potential applications in endoscopic procedures.

#### 3.2.2. Seals

The purpose of sealing is to create a reliable barrier at critical joining points using seals, preventing the intrusion of bodily fluids (such as gastric acid and blood), medical disinfectants, and external contaminants into the optical, electronic, and mechanical systems. It also ensures that the device maintains stable performance after repeated bending, twisting, and multiple sterilization cycles.

The commonly used seals for endoscopes are primarily medical-grade silicone rubber, which is widely applied in sealing insertion tube joints, working channel ports, and light-guide fiber interfaces. Fluoropolymers are suitable for sealing scenarios involving optical components at the distal tip and under highly corrosive environments. Thermoplastic elastomer (TPE) seals are often used for the housings of disposable endoscopes.

#### 3.2.3. Solders

In addition to the above-listed materials, solders are used for lower temperature bonding, when the base material does not melt. The term soldering refers to the group of joining processes that bond base materials together through a capillary penetration of a molten filler metal at temperature below the solidus of the base materials. The solder alloy has a liquidus temperature not exceeding 450 °C [93,94].

#### 3.2.4. Brazing Alloys

If the filler metal melting temperature exceeds 450 °C, it is considered brazing. The molten brazing filler alloys similarly penetrate the bond gap through a capillary action at a temperature above 450 °C but below the solidus temperature of the base material. A prefabricated coating called “bond pads” allows for the metallization of refractory metal and ceramic surfaces, making the brazing process easier.

#### 3.2.5. Electrodes, Wires, and Fluxes

If the filler material and the base materials melt simultaneously to form a molten pool, it is considered fusion welding. During fusion welding, filler materials commonly include welding electrodes, welding wires, and fluxes and shielding gases. In endoscope manufacturing, fusion welding is generally suitable for welding stainless steels, titanium alloys, nickel-based alloys, or dissimilar metals.

#### 3.2.6. Shielding Gases (SGs)

Shielding gases include inert gases and semi-inert gases. Among the noble gases, only helium and argon are cost-effective enough for welding applications. Pure argon and helium are used only for some non-ferrous metals. Semi-inert shielding gases, or active shielding gases, include carbon dioxide (CO_2_), oxygen (O_2_), nitrogen (N_2_), and hydrogen (H_2_). Most of these gases may damage the welds in large quantities, but when used in small, controlled quantities, they can improve welding arc or molten pool characteristics [94,95].

Common shielding gases include argon, helium, and carbon dioxide, which can be applied in pure form or as blends of two or three gases.

It can be summarized that the selection of joining materials and shielding atmospheres is highly dependent on the base materials employed and the functional requirements of inner/outer housings, ceramic substrates, and optical or structural components. Inert gas shielding is essential for preventing oxidation during the joining of reactive metals, while appropriate fillers ensure sufficient bond strength, biocompatibility, and stability under physiological conditions and repeated sterilization.

### 3.3. Joining Processes

**Figure 2:**
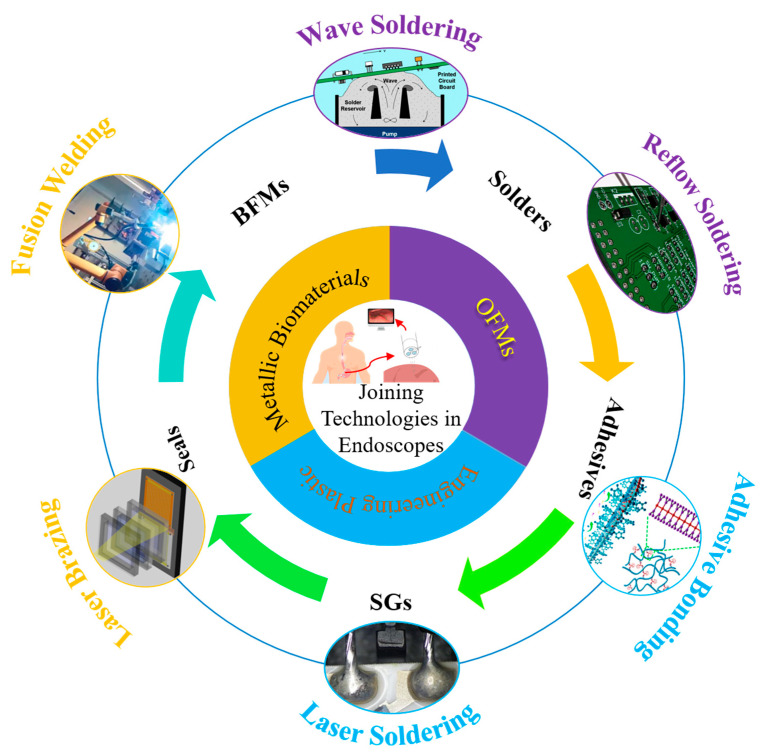
Schematic framework of joining technologies for endoscopes [42,100,101,102,103], illustrating the relationships among material categories, auxiliary consumables, solders, seals, adhesives, shielding gases (SGs), and typical joining processes. The circular flow highlights the sequential integration of dissimilar materials, consumables, and processes in precision endoscope fabrication.

As illustrated in Figure 2, the joining technologies for endoscopes are systematically organized in an interconnected framework, which shows the compound relationships between core material categories, auxiliary consumables, and typical joining processes. This schematic serves as an overview for the comprehensive analysis of each joining technology. Common joining methods include mechanical fastening (bolts, screws, rivets, and clamps), fusion welding, soldering, brazing, adhesive bonding, and mechanical interlocking mechanisms.

Welding is generally reserved for strong metallic bonding of two alloy pieces through melting and solidification of the molten pool, which mixes the base materials with the filler material. In contrast, sealing refers to the formation of a tight, leak-proof barrier between any materials, which blocks liquids, gases, and contaminants, thereby protecting internal components and ensuring the safety and reliability of the device.

#### 3.3.1. Adhesive Bonding

Adhesive bonding achieves material joining by leveraging surface adhesion, where the bonding strength originates from physical intermolecular interactions: polar/polarizable group interactions, hydrogen bonds, and van der Waals forces. It avoids the drawbacks of traditional welding, which can cause excessive damage to the base material, or mechanical joining methods that cannot provide a complete seal.

Curing systems include thermal curing (epoxy [104], polyurethane [105], phenolic resins, melamine resins, silica resins [106]), UV curing (UV-cured epoxy [107]), chemical curing [108] (polyurethane [109], silicone), moisture curing (silicate-based adhesives and sealants) [110], radiation curing [111], and curing by exclusion of oxygen (anaerobic adhesives) [112].

The CCD/CMOS image sensor is directly bonded to glass cover “A” for optical sealing [113]. Glass covers “A” and “B” are precisely joined to ensure imaging performance, with “B” adhesively fixed inside a stainless-steel imaging bracket. Adhesive around “A” further reinforces and seals the assembly. After focusing, the lens frame is bonded to the bracket to stabilize the objective lens unit.

Three adhesives (urethane acrylate, two-component epoxy, EPVP) were used for glass–glass and glass–stainless steel bonding [45]. The joints resisted 800 cycles of 121 °C steam sterilization. Key benefits: no glass metallization needed; adhesive ductility accommodates large thermal expansion mismatch between glass and stainless steel; residual shear strength of sapphire–stainless steel remains sufficient for use.

Precision joining is not inherent to general adhesive-bonding processes; however, the adhesive-bonding of CCD/CMOS image sensors to glass covers for optical sealing exemplifies precision bonding in practice. Such processes require micrometer-level positioning, rigorous cleaning, tight control of thin adhesive layer thickness and uniformity, and careful stress management to ensure optical and imaging performance. Furthermore, long-term reliability issues must be carefully mitigated, including adhesive yellowing or outgassing, and pixel drift caused by thermal stress.

#### 3.3.2. Laser Soldering

Laser is widely used in medical devices [114], and laser soldering, with its efficient joining characteristics, has become an important method for the precision joining of endoscopic base materials [115]. This technology uses a solder as an intermediate layer, and a focused laser provides a precise heat source to heat the target surfaces and melt the solder. The molten solder wets and spreads at the interface, and after cooling and solidification, a stable metallurgical joint is formed [116].

Historically, this kind of interlayer has been classified as either solder or braze alloy. The American Welding Society sets 450 °C as the boundary, defining interlayers with a liquidus temperature above 450 °C as brazing, while those below this temperature are considered soldering. However, there are different opinions: U.S. military specification MILSPEC uses 429 °C as the line [117]. Additionally, some engineers involved in electronics believe that only materials with melting points below 315 °C qualify as solder. In practice, most soldering in the electronics is completed at temperatures below 300 °C.

In the endoscope fabrication, Sn is one of the most used solder base in robotic laser soldering, and most solders contain Sn, while the most frequently used base material is Cu.

Jo et al. [118] fabricated Al FPCB/Cu FPCB lap joints, which were joined using SAC305 and Sn-57Bi-1Ag as solders by laser soldering. FPCB features a structure where Cu is printed on PI substrates, with the two layers bonded by a thermosetting resin adhesive [119]. How to evaluate the bond energy of the Cu/PI interface remains a challenge [120]. From Jo’s results in Table 4, the joints were prepared within 2 s. The proposed low-temperature laser soldering minimized embrittlement risks of the intermetallic compound (IMC) layer, resulting in excellent fracture energy in laser-soldered FPCB lap joints.

Based on the melting point of the solder, Puttlitz and Stalter [121] categorized the solders into the following four types:

(1) Low melting temperature (<180 °C), e.g., tin–bismuth (Sn-Bi) solders;
(2) Melting temperature is in the range of 180 °C to 200 °C, equivalent to eutectic Sn-Pb solder;
(3) Mid-range melting temperature (200 °C to 230 °C), lead-free solders;
(4) High melting temperature (230 °C to 350 °C), solders containing Ag, Cu, Pb, and Au.

Among the proposed solders [121,122,123,124,125,126,127,128,129,130,131,132], tin–copper (Sn-Cu), tin–silver (Sn-Ag), and tin–silver–copper (Sn-Ag-Cu) [121] exhibited excellent wettability. Sn-Bi series solders [122,123,124,125,126] have a low melting point, good electrical conductivity, and are suitable for low-temperature soldering, which can protect or seal heat-sensitive components.

Indium-based solders [127,128,129] can exhibit a very low melting point (156.60 °C) and can be alloyed with metals such as Sn, Bi, and Ag to form ultra-low melting point solders with melting points of 50–150 °C, suitable for soldering temperature-sensitive components. Their excellent wettability eliminates the need for flux during soldering, and their high ductility relieves thermal stress, improving joint reliability. Furthermore, indium-based alloys possess good electrical and thermal conductivity, ensuring stable signal transmission and effective heat dissipation. Through high-purity refinement and alloying, they meet the ISO 10993-1:2025 standard [98], making them suitable for components that contact the human body. In addition, the indium oxide film provides corrosion resistance and environmental stability, extending the service life of the device.

In the packaging of electronic components and heat-sensitive parts, traditional single-component solders have long faced the bottleneck of difficulty in simultaneously achieving “low-temperature soldering compatibility” and “high thermal and electrical conductivity”. This problem is particularly prominent in endoscope fabrication: the control chips and circuit boards of endoscopes are typical heat-sensitive components. Excessively high bonding temperatures can easily lead to chip performance degradation and circuit aging. At the same time, the heat generated during chip operation must be rapidly dissipated, and the soldered joints must meet the miniaturization and thinning requirements to fit the narrow internal space of the endoscope.

To overcome this limitation, Schmid and Doesburg [130] proposed a solution combining material innovation and manufacturing breakthroughs. The core involves constructing a “low-melting-point solder matrix–high-conductivity solder” composite with two designs: a mixed powder system of Sn- or In-based low-melting-point solder powders combined with Cu, Au, or carbon nanotube fillers; and a core–shell composite powder with a high-conductivity core and low-melting-point solder shell. A kinetic spraying process was used to prepare the composite solder coating, enabling low oxide content, high density, and precisely controlled thickness and composition. This approach resolves the low conductivity, excessive soldering temperature, and poor coating thickness controllability of traditional single-component solders.

Au-Sn solders may reduce reliability due to residual stress from soldering [131]. The stress arises from the mismatch in coefficients of thermal expansion (CTE) between the semiconductor laser and submount during cooling from the solder melting point to room temperature; soft solder relieves stress via deformation, while hard solder has little such effect. A protection mechanism and a hard solder, selected from the group consisting of AuSn, AuGe, and AuSi solder, have been investigated [132]. The as-soldered joints exhibit better tensile strength, but the soldered joints also have greater residual stresses. In addition, silver shows intrinsic antibacterial activity. Microbial growth at joint interfaces is suppressed during long-term in vivo application. The risk of biological contamination is reduced, and the device biocompatibility is enhanced.

It is suggested that a synergistic strategy be developed to combine thin-film engineering (evaporation, sputtering, electroplating) with silver anti-oxidation layers to reduce residual stress and improve sealing performance. Given the multifunctional requirements of endoscopic devices, two or more hybrid joining methods are generally required. Silver has a suitable melting point and high thermal conductivity. The low-thermal-input demand for precision endoscopic joining is thus satisfied, and thermally induced residual stress is minimized.

#### 3.3.3. Laser Brazing

In laser brazing, a filler material is placed on or between the contact surfaces of the materials to be joined. A laser is used to heat the filler, causing the filler material to melt while the base material does not melt. The molten filler material flows into the narrow gap between the contact surfaces through capillary action. When the filler material cools and solidifies, a diffusion bond is formed at the interface [133].

Compared to laser soldering, laser brazing involves higher temperatures, making it suitable for joining high-melting-point metals such as Cu/SS and SS/NiTi, and allowing the joints to withstand subsequent heating processes [134].

Endoscope manufacturers urgently require fabrication processes that enable products to withstand multiple repeated sterilization cycles without corrosion. Such devices are made of small, thin-walled metal tubes and sheets, which cannot be joined using adhesive bonding, brazing, or soldering. The introduction of laser heat sources has become a well-recognized ideal solution. It is suggested that laser brazing is suitable for joining load-bearing or high-temperature endoscopic components (e.g., SS/SS, SS/Ti alloys, SS/Al alloys, SS/sapphire, plastic/metal, ceramic/metal), but glass surfaces require prior metallization with a Ti/Au or Cr/Ni transition layer before brazing with gold-tin eutectic filler.

#### 3.3.4. Wave Soldering

Barnes, Elliott, and Strauss proposed the “bulk soldering with solder wave” [135] mode for the first time, addressing the current situation of printed circuit soldering at that time, which relied on “manual flux and overall dip soldering” or “roller transfer soldering”.

Prior to soldering, electronic components are inserted through the holes in the insulating panel via their pins, with the component bodies mounted on the upper surface of the panel and the pins protruding from the lower surface. During soldering, molten solder is ejected upward through a nozzle to form a stable wavy flow, which impacts the printed circuit side on the lower surface of the panel and the protruding pins. After being ejected, the solder surrounds the pins and ultimately bonds with the pins and the copper-coated areas on the insulating panel, forming firm as-soldered joints and ensuring effective electrical joining between the electronic components and the printed circuit.

#### 3.3.5. Reflow Soldering

Reflow soldering is a soldering process primarily used in surface mount technology (SMT) and is widely applied in the soldering of electronic components. In endoscope fabrication, it is primarily used for soldering circuit boards in various control systems. For purely surface-mount PCB, reflow soldering is more commonly used.

Reflow soldering and wave soldering, as two mainstream soldering technologies for electronic assembly, exhibit distinct applicability boundaries in endoscope fabrication. Reflow soldering [136,137,138] is mainly suited for the assembly of miniaturized, high-density electronic components, such as image sensors, microprocessors, and flexible printed circuits (FPCs) integrated in endoscope modules. Its controllable thermal profile minimizes thermal shock to heat-sensitive materials and low-melting-point solders (e.g., AuSn, SnBi). In contrast, wave soldering [139,140,141,142] is limited to non-critical, large-sized sub-assemblies, typically for through-hole components (THCs). Wave soldering is unsuitable for delicate distal tip components, as it may damage micro-optics, deform polymer housings, or cause excessive intermetallic compound (IMC) growth and solder bridging. Therefore, reflow soldering is the preferred technology for core precision components in endoscopes, whereas wave soldering is only used as a supplementary method.

#### 3.3.6. Other Fusion Welding Used in Endoscope

Fusion welding is classified according to the means of heating and pressure application, the type of filler material, and the equipment used. The most used welding methods include gas metal arc welding (GMAW), gas tungsten arc welding (GTAW), flux-cored arc welding (FCAW), shielded metal arc welding (SMAW), and submerged arc welding (SAW).

Fusion welding is particularly well suited for metal–metal joints, as it enables the formation of high-strength metallurgical bonds between similar or dissimilar components. By achieving complete melting and mixing at the interface, fusion welding produces robust, mechanically reliable joints with excellent load-bearing capacity and structural integrity, making it highly favorable for load-bearing structural components in endoscopic assemblies.

### 3.4. Requirements for Endoscopic Joining

Endoscopic joining must satisfy strict constraints:

(1) Low residual stress to avoid glass cracking or interface debonding;
(2) Sterilization resistance (autoclave, ETO, H_2_O_2_);
(3) Biocompatibility;
(4) Hermeticity;
(5) Thermal–mechanical stability under cyclic loads.

## 4. Joining of Components Used in Medical Endoscopes

In medical endoscopes, the image transmission component is a camera module, which includes ultra-fine electronic wires and a PCB. The copper pads of the PCB are subjected to electroless nickel immersion gold surface treatment, and the micron-scale thin layer is covered with hemispherical solder balls. The PCB laminate material covers the copper pads, and the board is equipped with a pad array containing multiple independent units. Square pads are also arranged on the back of each PCB.

Both the top and bottom pads of the PCB need to be soldered. As shown in Table 5 of References [143,144,145,146,147,148,149,150,151,152,153,154], the bottom pads of the PCB are soldered using SMT to attach two light-emitting diodes (LEDs) and a camera. To prevent the LEDs and camera already soldered to the bottom pads from deforming and detaching due to heat during the soldering of the top pads, Cai et al. [144] used laser ball-bonding technology with SAC305 as the solder to weld the core wires of the ultra-fine electronic wire harness to the PCB pads for signal transmission. The ultra-fine electronic wire harness consists of, from outside to inside: a PFA outer insulation layer, a tinned copper metal shielding layer, a polyester tape layer [145], a mesh silver-plated copper shielding layer (silver-plated copper wire, containing copper and silver elements), a PFA inner insulation layer, and the main core wires.

Studies have pointed out that the tool tip is usually made of stainless steel, titanium alloy, or ceramic materials, and can be connected to a stainless steel spring by spot welding [146]. In addition, a scheme has been proposed to arrange a metal film on the outer surface of the distal window and weld it to a corrosion-resistant metal outer tube, which relies on tight fusion welding [147]. However, such a welding method makes it difficult to detect defects in the welding layer, which may lead to the neglect of joint defects in mass production, and thus cause the endoscope window to lose its fluid-sealing performance after a period of use [148].

Studies have improved the poor sealing performance of vacuum brazing for SS/quartz joints by using a pre-metallization-assisted laser brazing process. This method realizes interface wetting via Ni, Au, and Cu transition layers on quartz, combined with SS tube cleaning and hard/soft brazing fillers. It significantly increases joint sealing strength and hermeticity, thus solving fluid sealing issues for endoscopic optical windows [149]. For lightweight joining scenarios involving metal–polymer and polymer–polymer combinations, corresponding studies have proposed filler-free spiral laser welding and pure laser welding, enabling seamless joining of cylindrical inner and outer nested components and polymer shells of capsule endoscopes, thereby avoiding risks of adhesive degradation [150,151]. A composite joining combining laser welding, rivet bonding, and brazing was developed to assemble over ten components to batch-produce complex structures [152]. Other studies have proposed an adhesive-free laser joining technique, facilitating seamless assembly of multi-components including endoscope flanges, stents, and vascular clips [153].

The key features of different joining processes are outlined as follows: (1) Brazing and laser welding form metallurgical bonds with excellent fluid sealing performance suitable for in vivo applications. (2) Brazing and laser welding support diverse material combinations, including similar and dissimilar materials. (3) Adhesive-free processes reduce material degradation and contamination risks in compliance with medical device regulations. (4) Adhesive bonding enables integrated multi-component assembly for efficient mass production of complex endoscopes. (5) Lastly, since adhesive bonding is needed for device integration, further development should focus on durability and sterilization impacts on adhesive bonds [154].

The existing technologies still have some limitations and these limitations should be future research targets: high process complexity—processes such as sapphire–glass pre-metallization and high-precision laser welding involve complicated steps; difficult defect detection—internal defects of brazed layers and spiral weld seams are difficult to detect; insufficient interface reliability—dissimilar joining is prone to thermal stress and interface embrittlement, resulting in inadequate fatigue performance and long-term service stability of joints.

## 5. Main Challenges and Future Work in Endoscope Joining

### 5.1. Main Challenges and Bottlenecks

Despite substantial advances in joining for endoscope fabrication, it still faces prominent challenges in miniaturization, material compatibility, long-term reliability, and clinical safety. These issues directly determine sealing performance, structural integrity, service life, and biocompatibility, especially under repeated sterilization cycles and long-term in vivo service [155].

Table 6 provides a critical comparative overview of current joining techniques, highlighting critical limitations, degradation behaviors and failure mechanism, and clinical reliability risks [156].

The current joining of endoscopic components is confronted with major challenges and bottlenecks, such as base material selection, dissimilar material joining, low-residual-stress joining, material packaging and reliability, aging, the balance between manufacturability and service life, joint resistance to bodily fluids and repeated sterilization, and the verification of clinical-grade durability. These mainly include:

(1) The widespread application of metallic biomaterials, OFMs and engineering plastics poses severe challenges to both similar and dissimilar joining. During dissimilar joining of different types of materials, the significant thermophysical mismatch will induce high residual stress, interfacial reactions, and possible cracks. Therefore, multi-material systems present considerable challenges and constitute a longstanding bottleneck in current research.
(2) The dissimilar joining between endoscopic components and the integrated multi-process joining pose significant challenges to endoscope fabrication. The dissimilar joining is prone to induce issues such as interfacial reactions, high residual stress, poor wettability, and cracks due to the significant thermophysical mismatch. These problems not only seriously damage the structural integrity and sealing of endoscopes but also may lead to the joint failing to meet the relevant medical standards.
(3) Third, achieving joints with low residual stress is another major challenge in endoscope fabrication. Low residual stress and high strength are contradictory under certain conditions. We classify the relevant joining strategies into four categories:
  - Low-temperature adhesives: The base materials of such joints are mostly optical materials, forming the optical system of the endoscope. They are mainly used for joining sensors (including cameras, lenses, and LED), such as fixing the lighting optical path, sensors, and other components on the supports inside the endoscope. Curing methods include UV, moisture, radiation, curing, etc. [104,109,110,111]. The residual stress of joints is extremely low, which can effectively avoid damage to optical components.
  - Soft solder with a melting point below 450 °C: Soft solder realizes the packaging of electronic components on PCBs, forming the control system of endoscopes and their sensors. This is achieved by wave soldering, reflow soldering, and laser soldering [118,144].
  - Hard solder (or brazing) with a melting point above 450 °C: Hard solder is mainly used in processes such as laser brazing, suitable for joining glass, sapphire, and other components to the SS shells, or realizing SS/quartz joining to achieve reliable assembly and sealing [149]. Pre-depositing a metal film on the surface of glass or sapphire, adopting soldering, or even employing adhesives is also an important alternative.
  - Fusion welding is suitable for joining endoscopic shells and tubes, forming the mechanical system of the endoscope. Welded joints have high strength, but the residual stress is relatively large [151,153].
(4) In endoscope fabrication, joining technologies face persistent challenges in balancing multiple key conflicts during both fabrication and service. The joining temperature should be matched with the service temperature, as lower heat input helps reduce residual stress and improve manufacturing efficiency but may cause joint failure because of low-melting-point solders. Joint constraint needs to fit the service load; higher constraint improves assembly accuracy but easily leads to stress concentration and cracks under repeated movement. A trade-off must be made between manufacturability and service life, since low-heat-input and low-strength joining methods simplify production but cannot meet long-life requirements.
(5) The last challenge is the degradation of endoscope caused by sterilization. While effective methods for eradicating microorganisms have been widely adopted, the inactivation of certain viruses remains a clinical burden. A prominent example of this is the challenge posed by viruses embedded within biofilms during endoscopic procedures. Furthermore, endoscope sterilization is prone to optical quality degradation, image-quality degradation [157], sudden lens failure due to mechanical or thermal shock during repeated use–sterilization cycles [19], mechanical degradation, adhesive band disintegration [158], rusting, discoloration, and aging of O-rings [41]. Besides endoscope sterilization, more studies focus on the medical equipment degradation. High-cycle sterilization (up to 1000 cycles) [159] significantly degrades the surface wettability, mechanical integrity, and interfacial bonding of silicone materials.

Inspection mainly covers three categories of endoscopic components: external components (control handle, valve housings, biopsy port, insertion tube, distal end adhesive bands, and distal tip); internal channel (biopsy port, bifurcation, channel body, bending, and distal end); and distal tip and bending section components (objective lens, light sources, elevator or ultrasound tip, and adhesive bands).

### 5.2. Future Work

Achieving low-stress joining of dissimilar materials is critical in fabricating high-performance endoscopes. Taking sapphire as an example, its bonding mainly involves two interfaces:

1. On one hand, it is joined with the SS tube at the distal end, forming a sapphire–SS joint, which ensures hermeticity, surface wear resistance, clear light transmission, and resistance to repeated sterilization cycles.
2. On the other hand, it is internally bonded with OFMs and sensing components, forming a sapphire–glass joint, which ensures distortion-free optical interfaces, low stress, and good light transmission.

#### 5.2.1. Dissimilar Joining Strategies for Multi-Material Endoscopes

First, a combined strategy of thin-film engineering (glass metallization) and a silver anti-oxidation layer is recommended to convert sapphire–SS joining into metal–metal welding.

Second, high-performance adhesives that do not require pre-deposited metallization or thin films on sapphire are also recommended. Such adhesives must be waterproof, since water is the main factor damaging adhesive bonds [156]. They must also resist sterilization, which is a strict requirement; in clinical validation, yellowing and failure of adhesives are the most common issues leading to unacceptable sterilization performance and non-reusability of endoscopes.

#### 5.2.2. Synergistic Mechanisms of Multi-Joining Methods

In the multi-process joining system (Figure 2), for dissimilar material combinations with significant differences in physical and chemical properties, high sensitivity to thermal damage, and requirements for sealing, flexibility, and low stress (e.g., metal–glass sealing), low-stress coating plus joining or adhesive bonding is suggested. For components subjected to loads during service (e.g., Ti-SS joints), fusion welding or laser brazing is recommended. For the packaging of electronic components on PCBs in sensors and control systems, wave soldering, reflow soldering, laser soldering, and other methods show more advantages.

It is essential to reveal the synergistic mechanisms of multi-joining methods. Current research on the sterilization resistance of endoscopes has mainly focused on sterilization effectiveness and the control of degradation and aging. It is recommended to clarify the performance changes of joints under long-term service, including bonding, sealing, and sterilization resistance, to provide a basis for the safe application of endoscopes in MIS.

#### 5.2.3. Laser-Based Joining Methods for Flexibility

Laser-based joining methods (fusion, brazing, braze-welding) can be applied to join numerous components of endoscopes. By adjusting laser power and scanning speed, a wide processing range from low-temperature to high-temperature joining can be achieved. Laser joining is highly flexible and can be readily integrated with robots to build an intelligent manufacturing system. In addition, laser welding can be conducted through glass without causing damage to the glass cover.

## 6. Conclusions

This review presents a comprehensive survey of joining technologies in endoscopic devices, integrating base material categories, consumables, and joining processes. The contributions include an analysis of the advantages and limitations of adhesives, solders, braze alloys, and fillers, and proposes multi-component integration as a promising route toward high-performance endoscopes.

A synergistic strategy combining thin-film engineering (evaporation, sputtering, and electroplating) with silver anti-oxidation layers is proposed to reduce residual stress, improve joint hermeticity, and achieve multi-material integration and functional assembly of endoscopic components via multi-joining methods.

For dissimilar material joints with large differences in thermal expansion coefficients, high sensitivity to thermal damage, and requirements of sealing performance, flexibility and low stress (glass–metal), “thin-film engineering + joining” or adhesive-bonding are adopted. For load-bearing structural parts that bear external loads and require high mechanical strength and structural rigidity, such as Ti–stainless steel joints, fusion welding or laser brazing processes are used. For the packaging of electronic components on PCB boards in sensors and control systems, wave soldering, reflow soldering, laser soldering and other methods are applied. Laser-based joining methods (fusion, brazing, and braze-welding) offer precise control of heat input, deliver reliable bonding quality, and minimize damage to heat-sensitive components in endoscopes. These techniques are suitable for multi-process joining in endoscope fabrication.

## Figures and Tables

**Table 1:** Classification and applications of biomaterials for endoscopic base materials.

Base Materials	Specific Materials	Components	Advantages and Applications	Refs.
Metallic biomaterials	Stainless steels (SSs) 1. 304/1.4301 2. 316/1.4401 3. 440C/1.4125 4. 17-4 PH	Endoscope sheath.	Evacuation of hypertensive putaminal intracerebral hematoma	[44]
		Outer Tubes; Mounting Rings; Adapters	Fabrication of joints via SS/borosilicate glass, SS/quartz glass, and SS/sapphire glass assemblies, using adhesive bonding/curing	[45]
		Shafts	Shafts are welded to eyepieces for structural integration	[46]
		Aspiration needles	Enhances sharpness while maintaining corrosion resistance	[47]
		Graspers, shafts, wire ropes, clamp jaws, biopsy forceps	Controlling the opening and closing of the tools and lens rotation requires high toughness and fatigue resistance.	[48]
		Biomedical implant	17-4 PH exhibits sufficient biocompatibility	[49]
		Restraining coil	Constrains SMA wires to control bending deformation	[50]
	Titanium alloys	Titanium alloy blades	GlideScope titanium video laryngoscopes	[51]
		Extraction devices	Extraction devices for cystolithotripsy: TiNi alloy (Ti–53.5–56.8% Ni, balance)	[52]
		Microforceps/microscissors	Microforceps and microscissors are fabricated from nickel–titanium wires for surgical applications	[53]
		Push rod	NdFeB magnetic cylinder, Ti-alloy, and SS abdominal end	[54]
		End effector	Main structures are fabricated by machining bidirectional grooves on Ni-Ti tubes for robotic bronchoscopes	[55]
	Monel (Ni-Cu)	Tubular parts of endoscopic apparatuses	Exceptional corrosion resistance: withstands the dual challenges of bodily fluids and disinfectant environments	[56]
	SMA (Ni-Ti)	Bending component	Multi-directional bending mechanism based on 3 SMA wires	[50]
	Co-Cr (CC)	Aspiration needles	CC exhibits higher hardness than SS, enabling sharper and thinner needle tips	[57,58]
	Ni-Co-Cr	Shaft	Super alloy is used to fabricate the shaft	[59]
	Aluminum alloys	Heat sink	A heat sink is made of aluminum	[60]
Optical functional materials	Sapphire glass	Infrared transmission window	Enables infrared light transmission for the endoscope	[61]
	Medical-grade sapphire glass	Transparent cover.	Transparent cover	[62]
	Optical glass	Aperture	Seal the aperture in a hollow transducer	[63]
	PMMA	Endoscope shell	Fabricates non-digestible PMMA shells	[64]
	Quartz fiber	All-fiber coupling system	NGC fiber/C-SMF fused joint, with an optical coupling efficiency of 95.2%	[65]
Engineering plastic materials	ABS	Funnel entrance Endoscopic holster	Cylinders are bonded to funnels using epoxy	[66]
	PC	Gastrointestinal neuroprosthesis	Consists of PFA-coated SS electrodes.	[67]
	PEEK	Capsule with electrochemical sensor, PEEK rod internal fixation	Spinal implants; capsule integrates internal components on a flexible polyimide substrate	[68,69]
	PPSU	Multi-lumen compliant catheter	Enables a compliant multi-lumen catheter structure	[70,71]

Note: SS: stainless steel; SMA: shape memory alloy; CC: cobalt-chromium; PMMA: polymethyl methacrylate; ABS: acrylonitrile butadiene styrene; PC: polycarbonate; PFA: perfluoroalkoxy alkane; PEEK: polyether ether ketone; PPSU: polyphenylsulfone.

**Table 2:** Filler materials and shielding gases for endoscopic joining.

Materials	Submaterials	Typical Materials	Components	Advantages and Applications	Refs.
Filler materials	Adhesives	Epoxy resin	Imaging optical components; Illumination light source components	It bonds the distal ends of the fibers, attaches the fibers to the sheath tubes, fixes lenses in the tube, secures the window assembly to the fiber distal ends, and attaches a prism to the sheath inner tube.	[88]
	Tissue adhesives	Cyanoacrylates Fibrin glues Thrombin	Hemostasis, wound closure, and fistula repair.	Collagen-based sealants, PEG polymers, and other similar adhesives, with potential applications during an endoscopic procedure.	[89]
	Seals	Epoxies	Support device	Seals the endoscope’s distal end.	[90]
	Solders, solder fluxes, solder pastes	Sn, Cu, Ag, and their alloys	Circuit board; Imaging sensor	Sensors are bonded to the circuit board. Copper film.	[91]
	Brazing alloys, brazing fluxes	Silver brazing	Flexible ultrasonic scalpels	Snare wire parts are brazed to the connection parts.	[92]
	Welding electrodes, wire, and fluxes	Sn, bond pads, deposited gold.	Stereo camera module, polygonal substrate, lens barrels.	The inner housing can be welded to ceramic substrates or placed on deposited gold and brazed to them. The outer housing is welded to the distal end.	[93,94]
Shielding gases Vacuum	Inert shielding gases	He	Al, Mg, Cu tubes, shells.	Helium and argon protect the molten metals from oxidation. Helium produces a hotter welding arc at higher voltages than argon.	[94,95]
		Ar			
	Semi-inert shielding gases	H_2_	SS, Al, Mg, Cu, Ti tube, shells.	Hydrogen improves molten metal fluidity but causes embrittlement in solidified welds. Small amount of oxygen can enhance arc stability and reduce molten metal surface tension. Nitrogen improves pitting corrosion resistance of steels. Carbon dioxide is a low-cost shielding gas that increases weld penetration but with greater spattering.	
		O_2_			
		CO_2_			
		N_2_			
	Mixed	Ar-CO_2_			
	Vacuum	–	–	The absolute pressure can reach 10^−3^–10^−2^ Pa.	

Note: SS: stainless steel; Mg: magnesium; Cu: copper; Ti: titanium; PEG: polyethylene glycol.

**Table 3:** Effects of sterilization mode on aging and degradation of endoscope adhesives.

Adhesives	Sterilization Mode	Aging	Strength Retention	Degradation Mechanism	Refs.
Optical (adhesive bonds, lenses, fiber bundles)	Repeated clinical use—sterilization cycles (Steam/ETO/H_2_O_2_, lifespan: 15–400 cycles)	Optical degradation, image quality decline, yellowing, interface microcracks, sudden lens failure	Significant strength loss, reduced optical transmission, shortened service life	Thermal/mechanical stress from repeated sterilization cycles, fatigue, interface debonding, and aging.	[19]
Polyurethane acrylate (GB 368, Delo)	Steam autoclaving, 121 °C/2 bar, 800 cycles	Slight surface yellowing, no obvious interface debonding or microcracks, excellent integrity retention	At 121 °C, the shear strengths were reduced by a factor of three to ten	Low water absorption, dense molecular chain crosslinking, superior thermal stability, interfacial mismatch.	[45]
Epoxy adhesive (M-31 CL, Henkel-Loctite)	Steam autoclaving, 121 °C/2 bar, 800 cycles (only glass–glass)	Slight yellowing at glass–glass interface, significant interface debonding and sharp strength drop for glass–SS		Incompatible mismatch.	
Endoscope adhesive/bonding interface	ETO	No obvious discoloration; long cycle may induce slight plasticization	Moderate decrease; bond strength gradually reduced with repeated cycles	ETO permeates into bonding interface, causes slight chain swelling and weakens interfacial adhesion.	[82]
	H_2_O_2_ sterilization	No yellowing, no cracking; compatible with most polymer adhesives	Slight reduction	Mild oxidation on adhesive surface, no deep molecular damage or interface separation.	
Vitralit^®^ 1655 (One-part epoxy, dual-cure)	Steam autoclaving (121 °C/2 bar, 800 cycles)/ETO	Slight yellowing, interface debonding in glass–SS, no obvious microcracks in glass–glass	Minimal effect on adhesive bond strengths	Low elongation, interfacial stress concentration under high temperature/humidity, thermal oxidation.	[96]
Vitralit^®^ E-7090 VHS F (UV-curable acrylate)	Steam autoclaving (121 °C/2 bar, 800 cycles)/ETO	Slight yellowing, no obvious interface debonding, good bondline integrity	Minimal effect on adhesive bond strengths	High elongation to accommodate mismatch, low water absorption, thermal softening at high temperature	

Note: autoclaving: steam autoclaving; ETO: ethylene oxide sterilization; H_2_O_2_ sterilization: hydrogen peroxide sterilization; standards: ISO 10993-1:2025 [98].

**Table 4:** Solders, melting point, and wettability.

Solder	Melting Point OR Range (°C)	Wettability	Refs.
SAC305 (Sn_96.5_Ag_3.0_Cu_0.5_)	217–220	Better	[118]
Sn-57Bi- 1Ag	140		
Sn-58Bi (eutectic)	138–139	Control group	[120,121]
Sn-58Bi-1Cu	-	Slightly	
Sn-Pb37	183	-	[121]
Sn-Zn9.0	198.5		
Sn-Cu0.7	227		
Sn-Ag3.5	221		
Sn-Ag3.0Cu0.5	217–220		
Sn-Ag2.0-Bi4.0-Cu0.5-Ge0.1	210–217		
Sn-Ag2.8-In20.0	175–187		
Sn-52In (soft solder)	118		
Sn-58Bi-2In	129.68	-	[122]
Sn-58Bi-2Al	142	Worse	[123]
Sn-xBi-3Zn (x = 37, 39, 41, 43)	136.7–138	Depends	[124]
Sn-58Bi-2Sb	147	Better	[125]
Sn-58Bi-0.5La	137.8	Superior	[126]
In-48Sn-20Au	152	Superior	[127]
In-48Sn-0.5Ag	113–117	Superior	[128]
In-20Sn-2Zn	–	Superior	[129]
Sn—Ag—Cu, Sn—Cu, Sn—Cu—Ni, Sn—Ag, Sn—Ag—Bi, Sn—Bi—In, Au—Sn, Sn—Zn, Sn—Zn—Bi, Sn—Bi—Ag, Sn (), Sn—In, In (156.60), In—Ag, Sn—Pb			[130]
Au-Sn (hard solder)	260–280	–	[131]
	270–280	–	[132]
AuGe (hard solder)	361	Au_88_Ge_12_ eutectic	
AuSi (hard solder)	360	Au_81_Si_19_ eutectic	

**Table 5:** Comparison of joining methods for medical endoscopes.

Material 1/ Component	Material 2/ Component	Welding Filler Materials Braze Alloys, or Solders	Welding, Brazing, or Soldering	Applications	Refs.
Distal end window (quartz, BK7, sapphire)	Outer tube (Monel^®^, VA)	Solderable ring-shaped metal layer and UV-curable adhesive	Soldering; Adhesive bonding; Soldering	End window + Outer tube	[143]
Camera module (LED, cameras)	Cu pads	SAC305	SMT	PCB for medical endoscopes (Camera module)	[144]
Electronic wire harness	Cu pads	SAC305	Laser tin-ball soldering	PCB for medical endoscopes	
Tool tip	SS spring	Sn	Spot welding	Endoscopic multifunctional biopsy forceps	[146]
The shaft (SS/Ceramics)	Eyepiece (Sapphire)	Metallized coating or glazing methods; Metallized	Tight fusion welding	Use in scenarios involving contact with bodily fluids	[147]
SS tube	Quartz sheet	1. Quartz is pre-treated with metallized nickel, gold, and copper; 2. Hard or soft solder.	Laser brazing or soldering	SS/quartz joints were vacuum brazed with organic adhesive at 800–900 °C	[149]
Cylindrical outer component	Coil-shaped inner component	—	Spiral laser welding	Weld the outer to the inner coil-shaped component.	[150]
Shell 1 (polymer)	Shell 2 (polymer)	—	Laser welding	The first and second outer shells are joined using laser welding	[151]
Spiral tube, outer tube end, connector, inner tube end, mesh tube, winding wire, outer sheath.		Adhesives	Laser welding; Rivet bonding; Soldering	Join a connector to the end of a flexible tube	[152]
Endoscope flange weld, pacemaker, housings and leads, welded wire stents, stent marker weld, sealing of glass tracers		—	Autogenous laser joining	Enables seamless component assembly	[153]
SS	Sapphire	Au or Au-contained solder	Gold-based soldering	Metallization on sapphire	[154]

**Table 6:** Comparative critical analysis of joining technologies: limitations, degradation mechanisms, and clinical reliability risks.

Joining Method	Critical Limitations	Degradation and Failure Mechanism	Clinical Reliability Risks
Adhesive Bonding	Chemistry-dependent; low stability; poor sterilization resistance	Autoclave-induced hydrolysis; interface debonding; small-molecule leaching	Gradual degradation after repeated sterilization; biocompatibility varies
Laser Soldering	Low heat resistance; easy oxidation; unstable IMC layers	Sn-based solder oxidation; brittle IMC growth; thermal fatigue fracture	Poor sterilization resistance; short service life for reusable endoscopes
Laser Brazing	Requires metallization; high residual stress; high cost	CTE mismatch cracking; brittle phases; stress relaxation failure	High cost; only for rigid parts; difficult for miniaturized modules
Reflow Soldering	For PCB/FPCB; thermal damage to CCD/CMOS	Polymer thermal degradation; pad lift; solder cracking	Risks imaging/sensor damage; not for distal optical parts
Wave Soldering	For through-hole PCB only; high thermal shock	Uneven wetting; oxidation; solder bridging	Only for non-critical circuits; incompatible with sensing modules
Fusion Welding	High heat input; large distortion; burn-through risk	Grain coarsening; residual stress; hot cracking	Damages precision parts; unsuitable for miniaturized/optical components

## Data Availability

Not applicable.
